# Routinely collected data for randomized trials: promises, barriers, and implications

**DOI:** 10.1186/s13063-017-2394-5

**Published:** 2018-01-11

**Authors:** Kimberly A. Mc Cord, Rustam Al-Shahi Salman, Shaun Treweek, Heidi Gardner, Daniel Strech, William Whiteley, John P. A. Ioannidis, Lars G. Hemkens

**Affiliations:** 1 Basel Institute for Clinical Epidemiology and Biostatistics (CEB), Department of Clinical Research, University Hospital Basel, University of Basel, Spitalstrasse 12, 4031 Basel, Switzerland; 2 Centre for Clinical Brain Sciences, University of Edinburgh, Edinburgh, EH16 4SB UK; 3 Health Services Research Unit, University of Aberdeen, Aberdeen, AB25 2ZD UK; 4 Institute for History, Ethics and Philosophy of Medicine, Hannover Medical School, 30625 Hannover, Germany; 5 Stanford Prevention Research Center, Department of Medicine, Stanford University School of Medicine, Stanford, CA 94305 USA; 6 Meta-Research Innovation Center at Stanford (METRICS), Stanford School of Medicine, Palo Alto, CA 94304 USA; 7 Department of Health Research and Policy, Stanford University School of Medicine, Stanford, CA 94305 USA; 8 Department of Biomedical Data Science, Stanford University School of Medicine, Stanford, CA 94305 USA; 9 Department of Statistics, Stanford University School of Humanities and Sciences, Stanford, CA 94305 USA

**Keywords:** Routinely collected health data, Electronic health records, Registries, Evidence-based medicine, Trials, Clinical epidemiology

## Abstract

**Background:**

Routinely collected health data (RCD) are increasingly used for randomized controlled trials (RCTs). This can provide three major benefits: increasing value through better feasibility (reducing costs, time, and resources), expanding the research agenda (performing trials for research questions otherwise not amenable to trials), and offering novel design and data collection options (e.g., point-of-care trials and other designs directly embedded in routine care). However, numerous hurdles and barriers must be considered pertaining to regulatory, ethical, and data aspects, as well as the costs of setting up the RCD infrastructure. Methodological considerations may be different from those in traditional RCTs: RCD are often collected by individuals not involved in the study and who are therefore blinded to the allocation of trial participants. Another consideration is that RCD trials may lead to greater misclassification biases or dilution effects, although these may be offset by randomization and larger sample sizes. Finally, valuable insights into external validity may be provided when using RCD because it allows pragmatic trials to be performed.

**Methods:**

We provide an overview of the promises, challenges, and potential barriers, methodological implications, and research needs regarding RCD for RCTs.

**Results:**

RCD have substantial potential for improving the conduct and reducing the costs of RCTs, but a multidisciplinary approach is essential to address emerging practical barriers and methodological implications.

**Conclusions:**

Future research should be directed toward such issues and specifically focus on data quality validation, alternative research designs and how they affect outcome assessment, and aspects of reporting and transparency.

## Background

Routinely collected health data (RCD), such as electronic health records (EHRs), registries, or administrative claims data, are useful for randomized controlled trials (RCTs), especially those whose aim is pragmatic. RCTs embedded in routine data collection might be the next disruptive clinical research technology [1]. However, numerous fundamental questions have recently been raised [1–8]. In this review, we summarize the promise and potential barriers, followed by methodological implications and research needs, for the better use of RCD for RCTs, thus collating an overview of the current applicability and promise of the use of RCD in clinical trials.

### Potential value of RCD for RCTs

**Fig. 1:**
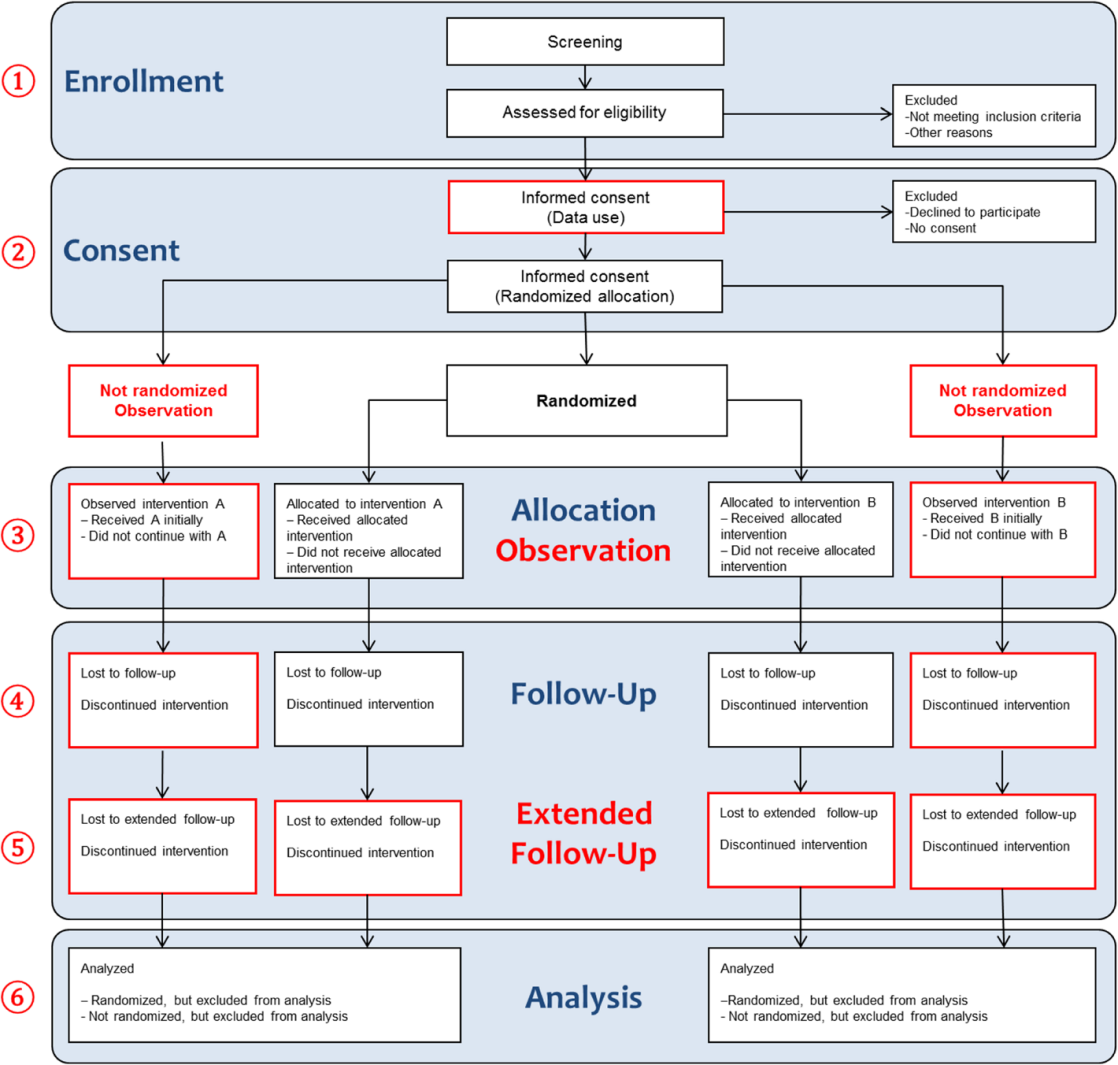
The role of routinely collected health data (RCD) in randomized controlled trials in various phases of a clinical trial (based on the Consolidated Standards of Reporting Trials [CONSORT] flow diagram [11]). (1) During enrollment, RCD sources can be screened retrospectively for eligible patients, but they can also be used prospectively as targeted screening and recruitment tools. (2) Informed consent could be given both for data use and for trial participation, so that when patients decline to participate in the randomized component of the trial, their usual care can still be followed. (3) Allocation can be facilitated by RCD through point-of-care randomization. Patients who are not allocated to an intervention but select care on the basis of personal and clinical preferences can be observed with RCD. (4) During the follow-up phase, RCD allows patients who would otherwise be lost to follow-up to be tracked, and thus less missing data may be encountered. (5) Long-term follow-up, such as in registries, may be possible with RCD even after formal completion of the primary study phase. (6) RCD allows analysis of both nonrandomized and randomized patients and direct supplementation of information to the randomized part of the trial

RCTs are often very expensive. Some trials are stopped early because of failure to recruit; some fail to generate useful evidence for clinical practice; and in some, the results are not disseminated at all. Various limitations of RCTs are used as arguments to support observational “real-world” RCD studies [9, 10]. We argue that some of the limitations of RCTs are better addressed with RCD within a randomized design, avoiding the problems of confounding when assessing treatment effects (Table 1). The use of RCD can replace or supplement some or all procedures of traditional trials, and sometimes a blend of routinely collected and actively collected data may be more feasible and useful. In Fig. 1, based on a modified CONSORT (Consolidated Standards of Reporting Trials) [11] trial flow diagram, we illustrate the roles of RCD during the subsequent phases of a trial.

**Table 1:** Common limitations of randomized controlled trials and whether they can be amended by routinely collected health data

Limitations of RCTs [10]	What using RCD for RCTs can offer	Challenges	Potential of RCD to improve RCTs
Generalizability and real-world relevance	No specific data collection processes (follow-up visits, measurements) outside routine care, avoiding artificial situations	Random allocation of interventions may still require some deviation from routine care processes (e.g., obtaining informed consent).	Very high
Costs and resources	No costs to the trial for data collection processes and related activities (study site setup, study staff salary, monitoring and auditing activities, training costs)	Potential costs for obtaining the RCD (if the collecting entity does not provide it for free; e.g., data brokers); additional costs for data management, processing, merging, cleaning, and so forth	Very high
Specific conditions/subgroup effects	Larger sample sizes that are less influenced by resource constraints and feasibility issues may provide sufficient power for evaluating subgroups.	More opportunities for exploratory analyses with spurious findings	High
Late outcomes	RCD can provide long-term outcome data without actively following patients and often reducing the number of patients lost to follow-up	Patients moving away from RCD infrastructure will be lost and may still require active contact, highly dependent on RCD infrastructure	High
Speed	No cumbersome outcome ascertainment (follow-up contacts, data recording and collection) and no need for setting up the data collection infrastructure, thus results can be obtained faster	Management, processing, merging, and “cleaning” of large datasets may be time-consuming. Reporting of specific adverse events may be delayed.	High to moderate
Conflicts of interest/sponsorship bias	Collection of RCD is more objective and less easily manipulated to obtain a desired result.	Data may still be analyzed and reported nonobjectively to convey preferred conclusions.	Moderate
Understudied healthcare questions	Providing information on routine care allows researchers to address understudied healthcare questions because more resources are spared or different outcomes are collected.	Not all desired endpoints might be available; funding may not be the sole barrier	Moderate
Regulations	Obtaining approval for intervention imposes several bureaucratic loopholes; RCD are already available and might require different ethical clearance.	RCD still require approval in terms of data protection and confidentiality.	Moderate
Rare or uncommon conditions	Recruiting an appropriate sample size may be hard with rare diseases; larger samples with RCD and easier EHR or registry recruitment can reduce these difficulties.	Only possible if RCD resources are extensive, highly dependent on RCD infrastructure	Moderate

*Abbreviations: EHR* Electronic health record, *RCD* Routinely collected health data, *RCT* Randomized controlled trial

RCDs may make RCTs easier and more feasible by reducing costs, time, and other resources. This might mean larger RCTs for the same cost or RCTs in research areas where high costs and insufficient funding previously precluded their conduct. Finally, even when cost and resource limitations do not exist, RCD may foster novel research activities, such as the use of registries for rapid, consecutive trial enrollment [3, 4].

#### Value through better feasibility

Effective recruitment is necessary for a successful trial [12]. Targeted screening strategies to identify eligible patients with routine data may lead to more efficient recruitment. They may be used alone but also as a supplement to traditional methods. Researchers can screen electronic databases and contact eligible patients or their healthcare professionals, reducing costs associated with recruitment during the delivery of healthcare, sometimes for hefty fees [13]. Data-mining tools implemented in preexisting EHR systems can scan patient charts to identify eligible patients automatically; electronic chart alerts can then prompt the physician to suggest participation during a routine clinical encounter or through contact via a letter [14]. Registries of medical conditions, drug therapy, or devices are especially valuable, particularly when patients with rare diseases or other uncommon characteristics are sought [15]. Registers of individuals interested in research (see, e.g., www.registerforshare.org) that can be linked to EHRs also support pretrial identification of potentially eligible participants. Even more widely available than registries, health insurance databases provide an extensive sampling frame for patient recruitment, as well as a wealth of outcome data [5].

All or some outcome data could be taken from RCD, reducing the need for cumbersome follow-up visits, bespoke data collection, costly monitoring, and audits. Building new infrastructures outside standard healthcare, training research staff, or purchasing additional equipment are avoided. This may accelerate trial setup, provide faster results, and also reduce trial costs significantly. Site monitoring accounts for 9–14% of total trial costs [16]. In addition, administrative burden and staff costs account for 15–22% of the traditional total trial expenditures [16]. Most issues detected by monitoring are due to poor source documentation [17] (i.e., a data point is not inserted in the trial master file or a consent form is not properly filled).

#### Value through expanded research agenda

Research questions not otherwise amenable to trials (e.g., in rare diseases) might be answerable with RCD. For example, local and national registers of people with myotonic dystrophy played an important role in the successful recruitment strategy of the OPTIMISTIC trial [18].

Using RCD may help to address some traditional imbalances in the evidence landscape and reduce traditional research agenda biases. Treatments that are typically not championed by commercial interests, such as exercise or physical therapy, speech therapy, psychotherapy, or surgeries, are less supported by randomized evidence than drugs or devices. Any cost reduction could facilitate trials for interventions that typically strongly depend on public funding structures and noncommercial research support. By saving resources elsewhere, RCD-based trial research may broaden therapeutic options or even reveal better treatments.

For drug therapies, the use of RCD may allow independent realization of notoriously lacking head-to-head comparisons and evaluation of “blockbuster drugs” in pragmatic megatrials [19]. Those drugs are used by millions of individuals, but RCT evidence to support them comes only from several hundred or a few thousand patients, often without patient-relevant outcomes and with strict eligibility criteria. The possibility of long-term outcome assessments makes RCD an excellent tool for postmarketing surveillance. Public funders may also have more chances to initiate independent research, increasing transparency and potentially directly addressing areas with suspected publication or reporting biases. The conducted RCTs may better reflect the true healthcare needs and avoid “cost and convenience” biases resulting from choosing a research question on the basis of what is affordable.

Whereas many outcomes that are traditionally of interest in clinical research, including biomarkers and patient-reported outcomes, are not included in most RCD sources, RCD typically include outcomes that are not included in many traditional RCTs (return to work, need for home nursing, sick days, disability, and major events such as cancer diagnoses or accidents). Implementing RCTs at the point of care, with randomization occurring directly in EHR platforms, might lead to RCTs having more generalizable results that assess more patient-relevant and clinically relevant outcomes [6, 20, 21]. Such RCTs could provide insight in situations where surrogate or combined outcomes are often used for convenience or safety reasons but are considered subpar [22, 23]. RCD-based RCTs often have more patient- and clinician-relevant outcomes that can inform comparative effectiveness research and guide clinical decision-making rather than provide information for mechanistic or proof-of-concept studies [21]. With increasing incorporation of patient-reported outcomes and even mechanistic data (e.g., genomics) in EHR in routine care [24], this gap may eventually be removed. Indeed, increasing the research use of RCD may lead to changes in the outcomes collected in routine data, a process that needs to maintain a careful balance between workload and utility.

#### Value through improved design and data collection options

Instead of inviting a patient for a repeated measurement or calling his/her healthcare provider for the patient’s clinical information, the researcher can access the RCD database and extract it autonomously, which avoids disrupting the usual care environment and without coming to the attention of the patient or care provider or requiring additional work from either. By reducing the need to affect the flow of routine care and the need to contact patients and care providers, such as by artificial blinding and outcome assessment procedures, observer bias (i.e., Hawthorne effect) is minimized. This may be especially true for behavioral interventions [25].

Administrative databases offer a wider array of variables of interest to use in an RCT, including social factors, unemployment or disability status, or healthcare use. For example, an insurance claims database could be queried automatically at admission to identify individuals frequently visiting an emergency department to target them for a discharge-planning intervention.

Retrospectively linking RCT databases with RCD supports data collection after regulatory approval is given for a drug or device. For example, data from large approval trials could be linked with cancer registries for evaluation of postapproval safety concerns, or very long-term trial outcomes could be collected from registries, as was done in the West of Scotland Coronary Prevention Study [26].

### Practical barriers to using RCD for RCTs

Greater use of RCD in RCTs is challenging. When using RCD to overcome some of the limitations of traditional RCTs, several additional barriers may occur and can be classified into four principal domains: data, regulatory and ethical aspects, costs, and novelty (Table 2).

**Table 2:** Barriers in the use of routinely collected health data for randomized controlled trials and options for improvement

General barriers or issues	Pressing questions	Possible solutions, actions and additional comments
Data		
▪ Availability ▪ Management ▪ Linkage ▪ Accuracy ▪ Validity	▪ Is the desired outcome variable or RCD source available? ▪ Will it be possible to achieve the same data quality and accuracy with RCD as in traditional trials? ▪ Is the data linkage and management feasible in institutions with limited IT infrastructure?	▪ A central register of databases available for clinical trial research would be helpful, ideally with details about data quality. ▪ Establish core outcomes and structured outcome assessments in routine care ▪ Create RCD trial guidelines and RCD source validation guidelines to help standardize their use and reduce sources of bias or uncertainty ▪ Increase IT presence (particularly data analysts) to health research teams ▪ The more RCD is sought out and used in research, the greater is its availability and differentiation.
Regulatory and ethics		
▪ Collecting and obtaining the data ▪ Using and sharing the data	▪ What type of release must be given by the patients before their data can be collected or shared? ▪ Is it ethical to use RCD without asking for their permission, even if their data are anonymized? ▪ Can this data be considered of value and morally be sold? ▪ How are concerns about privacy and informed consent approached (particularly in the context of population-wide trials or Zelen designs)? ▪ Are data safety standards applied to RCD just as strictly as they are to traditional actively collected data? ▪ Who is responsible for the safety of the data?	▪ Ethical guidelines specifically regarding the collection and dissemination of RCD should be developed. ▪ Ethics and approval committees should deepen their knowledge of these novel ethical challenges. ▪ Whereas personal data are collected daily from many sources (e.g., phone use), collection, storage, and dissemination of data related to health require more definite ethical oversight and greater transparency to the general public. ▪ After safety issues are defined, researchers and stakeholders must ensure that data are safely handled, with full transparency of access.
Costs		
▪ Obtaining the data ▪ Managing the data	▪ Will data collectors (e.g. health insurers) share their data? Freely or at a cost? ▪ Is a constant increase in the generation of routine data really reducing the overall trial costs if the same institution collected the data in the first place? ▪ When is the use of RCD cost-effective?	▪ The financial worth of health data is not defined or explored; empirical data are necessary to determine the cost of both producing and maintaining health data ▪ Health data are already legally sold to many industries, and regulations/legislation must catch up with this aspect.
Novelty		
▪ Bureaucratic obstacles ▪ Unawareness ▪ Training to generate, collect, prepare, manage and analyze RCD for trials	▪ Will approval committees understand the implications of using RCD sources for clinical trials? ▪ What are the challenges that can be expected bureaucratically because most submission templates do not assume the use of RCD and absence of patient contact? ▪ Are data anonymization techniques clear? ▪ What training is required to qualify individuals who generate, collect, prepare, and manage RCD for clinical trial research?	▪ Develop, in collaboration with approval committees, RCD-specific templates and submission forms, especially in such studies where no patient contact is foreseen and therefore speedy approval is desired. ▪ Educate regarding data anonymization and confidentiality risks ▪ Include the concept of using RCD for RCT in clinical research education and teaching ▪ Create and use reporting guidance specifically for RCD-RCTs

*IT* Information technology, RCD Routinely collected health data, RCT Randomized controlled trial

### Data

Even when the RCD necessary to answer a research question is available, it may be difficult to locate and access. The data owner may not be easy to contact, may not be willing to provide or share the data, or may not be able to provide it in a form that one may need to conduct an RCT, such as aggregated data being offered when individual patient data are what is needed.

The datasets may be very large, requiring a substantial information technology (IT) system, including human resources, hardware, and software to sort through and organize the data in such a way that it can then be analyzed. Connecting or linking to a research database with a system that is either continuously collecting the data (such as an EHR) or to another database (such as insurance claims database) requires significant planning and software development.

A few RCD variables and some RCD source types may be more accurate and better validated than others. Each variable for each source has variability in its accuracy that makes it difficult to make a general accuracy judgment. Hence, different EHRs or registries may have different data quality (quantity of missing data as well as actual correctness of the data), but the major obstacle remains the variability within the same source [2, 7]. However, a validation of the RCD source by manually checking a sample of the dataset before each trial would become cumbersome and may offset the advantages of RCD use in the first place. Even with randomization, the quality of the data may sometimes still depend on the assigned intervention and thus may be different between the comparison groups.

All in all, each research question, or even each outcome estimate, should be carefully examined, paired with the specific RCD source and variables used, to establish whether such elements were appropriate and what degree of confidence can be placed in such outcome assessments. A population registry based on a unique identifier that every individual receives at birth and has been established for many years with considerable resources for quality assurance (e.g., in Denmark [27]) is likely more accurate than EHRs of a small commercial practice. Systematic validation standards clearly describing and comparing validity and accuracy of codes and algorithms used for identification of patients, conditions, treatments, or outcomes are currently not universally established for RCD.

### Regulatory and ethical aspects

Core ethical principles for clinical research include informed consent, independent ethics review, confidentiality, or risk management (e.g., audit, serious adverse event reporting). Although the principles themselves remain the same, differences exist in the way in which they can and should be applied in research with RCD. Some ethical issues, such as confidentiality, can become more significant, whereas others, such as consent and audit, might be simplified. In particular, when variations of usual care are explored, privacy-related issues typically dominate ethical assessments. Recent guidelines [28] and reports [29] addressing research with collected and linked health data highlight the opportunities and challenges of innovative and feasible concepts for consent and further oversight. Whereas some argue that even a “no-consent” model whereby patients would be unaware of participating in an RCT could be in line with ethical principles and current law [30], others advocate for the so-called integrated verbal consent models that incorporate a notification of randomization into the usual clinical discussion between physician and patient [8]. Although in recent public surveys the majority of the community still preferred written consent prior to participating in pragmatic RCTs [31], most would also accept verbal consent or general notification if written consent would make the research too difficult to carry out [32].

Templates for broad consent texts have already been developed and implemented for research with human biospecimens and might be applied in a modified and simplified version for research with RCD [33, 34]. Consistent with international ethical guidelines, ethics review committees may also waive the requirement for informed consent when research participation involves no more than minimal risk and requiring informed consent would make the study impracticable.

When using high-dimension datasets, effective anonymization is often quite difficult [35]. With a larger sample size, anonymity may be easier to achieve, whereas more detailed data may allow easier breach. The most appropriate data protection model, therefore, needs to be tailored to the individual RCD project. In general, research staff with access to confidential records must be adequately trained, and a liability protection considering patient privacy and potential data breech should be considered.

At a policy level, public and patient involvement builds another cornerstone for long-term public trust in research with RCD, especially when such research includes consent waivers or broad consent [29, 36]. Public interests, however, reflect not only the protection of privacy but also research with RCD that can improve public health. Overall, the uses of RCD, in particular its collection, storage, and dissemination, raise novel ethical considerations that may require further development of regulations to ensure adequate protections but without unduly constraining the potential benefits of greater research use of RCD.

### Costs

Setting up infrastructures to implement use of RCD for clinical research may be associated with enormous overhead costs. Specific investments may be needed before starting such research. Although the costs related to maintaining the RCD source (e.g., insurance claims databases) may not rely on the researcher, this should be considered in institutions where both clinical practice and research take place, such as in university hospitals. It may become common practice to charge for the release of RCD once it becomes more widely used. Alternative models involving supported access to RCD are also possible; Scotland’s electronic Data Research and Innovation Service provides access and support and publishes charging structures [37]. Even if data are shared for free, costs are associated with finding the correct data, negotiating its acquisition or access, and transferring or linking such data to the trial database. Specifically trained personnel and specific resources may be required to manage and link the data, as well as to ensure privacy and data protection. Once a trial database is established and linked to the RCD source, maintenance costs may be incurred. Nonetheless, it may be argued that many of these investments will be offset by later cost savings when RCD is used in trials (e.g., by making some monitoring activities obsolete). The real challenge will arise when costs and savings are borne and won by different organizations.

### Novelty

The novelty of using RCD for trials may itself be a barrier. Established structures, such as templates for ethical approval or grant proposals, are often not yet designed to apply to this kind of research.

Guidelines for use and handling of RCD often stem from nonexperimental research with other foci. For example, on one hand, the most widely used reporting guideline for this type of data was developed for observational RCD analyses (the REporting of studies Conducted using Observational Routinely collected health Data [RECORD] statement [38]), but there is no reporting guideline addressing the specific issues of RCD in the context of RCTs. On the other hand, there are initiatives to provide guidance, such as the recently drafted guidance for industry on approval of medical devices by the U.S. Food and Drug Administration [39].

Furthermore, the novelty of the technology itself will require additional training and data science staff necessary to implement RCD-RCTs embedded in routine care. Although RCD-RCTs may reduce the costs associated with training research staff for patient recruitment or outcome ascertainment, any savings may be offset by new expenses for training those who generate and collect the RCD so that the data can be used for research and for training researchers to prepare, manage, and analyze this data within a clinical trial framework.

### Methodological implications

In addition to general barriers to using RCD in clinical trial research, novel methodological problems and potential biases may be introduced. However, use of RCD may also reduce and preemptively avoid some internal validity biases and provide valuable insights into external validity by showing potential differences between included patients and/or nonincluded but eligible individuals.

RCD-based research obviously requires reasonable data quality, but this holds for both randomized and observational research using RCD. Data quality issues, including misclassifications, are much less of a problem with randomization, however, because this typically rules out the possibility that the explored intervention is related to data quality. This is in sharp contrast to observational studies, where determination of exposures may actually be strongly associated with data quality and increase risks of misclassification and detection biases. However, even in a trial, it may be problematic when the measurement of outcomes is associated with the allocated intervention. Bias might occur, for example, when one study intervention leads to more contact with healthcare professionals who collect the routine outcome data in a different way (e.g., by using more sensitive diagnostic procedures, by coding the data differently, or by using different time schedules for examinations). Possible solutions include standardized documentation of core outcomes (e.g., through a structured assessment of all patients at hospital discharge) and training of healthcare professionals to perform standardized data entry. Efforts at standardization may escalate cost, however, and diminish the advantages related to the ease and low cost of using the RCD.

Not only quality but also timeliness deserves attention, because timely assessment of safety issues may be challenging when a specific adverse event data collection mechanism is not in place as in traditional RCTs [7]. Because routine data are typically collected only at the time of clinical encounter and then need to be processed, registered in the database, and made accessible to the researcher, there may be substantial delay between occurrence of adverse events and recognition by the researchers. Combining routine data with active collection in a hybrid approach may help, for example, by performing telephone checks to randomized patients to seek adverse event information [7]. Active collection, however, requires substantial resources.

However, outcome data collection in RCD-RCTs may have advantages because it is often formally blinded, as in any traditional trial, with blinded endpoint assessment. Then, and when outcome data collection is standardized and unrelated to the intervention, any misclassification would be completely at random and only introducing noise and decreasing precision of outcome estimates.

Dilution of effects due to imprecision and misclassification may gain particular importance for noninferiority questions or evaluation of some adverse events, which may be less adequately addressed with RCD of uncertain data quality. One potential solution is to increase sample size to account for the increased noise that RCD brings. In principle, at least, easy provision of larger sample sizes is one of the key advantages of RCD-RCTs, so making this a routine requirement ought not to be a substantive barrier.

Data completeness of RCD-RCTs is not necessarily a problem; actually, sometimes it is quite the opposite, with levels of completeness that are rare in traditional trials. For example, the TASTE trial (Thrombus aspiration during ST-segment Elevation myocardial infarction) [40], embedded within the Swedish Coronary Angiography and Angioplasty Registry, evaluated more than 7244 participants with zero patients lost to follow-up. Internal validity may be compromised when mechanisms lead to loss to follow-up and missing data are not completely at random. RCD may shed light on this, because often there are still data collected for those patients even after dropout. RCD may in fact provide excellent information on whether a treatment is well-tolerated and by whom it is not, as well as on the intervention’s side effects or drawbacks. Furthermore, one can examine the outcomes of patients who deviated from the original treatment plan, such as patients who discontinued taking the allocated drug and had surgery instead. With an expanded RCD source such as a national EHR system, outcomes can be available even for those patients who were lost to follow-up. However, this is possible only when the RCD data sources are accurate and extensive enough (such as in Sweden [41] or Canada [42]) to track withdrawn patients.

### Next steps and research needs

Careful evaluation of data accuracy, including validation and clarification of algorithms, appears to be one of the most important issues. Other important questions may be asked. Are outcome estimates different when measured in RCD-RCTs compared with RCTs with traditional active data collection? If so, are they source-specific, or do they depend on the type of outcome? How can users of trial research determine if the data are sufficiently accurate? A central register listing routine datasets available for trial research, including information on data quality and validity, would be helpful. A general standardization of routine data collection to ensure that it is useful not only for patient care and administration but also for research would be desirable. Employment of electronic algorithms that could be used to automatically perform validation checks (either at the moment of data entry or as random, systematic, and regular checks) might also be helpful [7].

Other questions that require exploration are related to patient recruitment and consent. Does pragmatism affect the estimates of treatment effects? Are different consent models needed? Are Zelen trials done without obtaining consent from each and every participant, giving results similar to those of other trials that require consent from everyone? And how can randomization become a standard usual care procedure despite short appointments and constrained resources in clinical care?

Development of guidelines for review, conduct, and reporting of trials using RCD may be helpful. Systematic reviewers, health technology assessors, and regulators and other users of this research may need novel tools and some training to assess the quality and risk of bias of such evidence.

## Conclusion

RCD have substantial potential for improving the conduct and reducing the costs of RCTs. Future research should specifically focus on data quality validation, alternative research designs and how they affect outcome assessment, and aspects of reporting and transparency.Many of these issues will require multidisciplinary research efforts and a large international research initiative on RCD for RCTs. This will allow researchers to exchange, collaborate, and learn but would require support by some structured funding and resources. Overall, better understanding of how to make the best use of RCD for RCTs is needed.

## Abbreviations

CONSORT: Consolidated Standards of Reporting Trials
EHR: Electronic health record
IT: Information technology
RCD: Routinely collected health data
RCT: Randomized controlled trial
RECORD: REporting of studies Conducted using Observational Routinely collected health Data statement
